# Deep learning for evaluation of microvascular invasion in hepatocellular carcinoma from tumor areas of histology images

**DOI:** 10.1007/s12072-022-10323-w

**Published:** 2022-03-28

**Authors:** Qiaofeng Chen, Han Xiao, Yunquan Gu, Zongpeng Weng, Lihong Wei, Bin Li, Bing Liao, Jiali Li, Jie Lin, Mengying Hei, Sui Peng, Wei Wang, Ming Kuang, Shuling Chen

**Affiliations:** 1 Department of Gastroenterology, the First Affiliated Hospital of Sun Yat-Sen University, Guangzhou, Guangdong China; 2 Department of Medical Ultrasonics, Institute of Diagnostic and Interventional Ultrasound, the First Affiliated Hospital of Sun Yat-Sen University, No. 58, Zhongshan 2nd Road, Guangzhou, 510080 Guangdong China; 3 Clinical Trials Unit, the First Affiliated Hospital of Sun Yat-Sen University, Guangzhou, Guangdong China; 4 Department of Pathology, the First Affiliated Hospital of Sun Yat-Sen University, Guangzhou, Guangdong China; 5 Department of Liver and Pancreatobiliary Surgery, Dongguan People’s Hospital, Dongguan, Guangdong China; 6 Department of Liver and Pancreatobiliary Surgery, Shunde Hospital of Southern Medical University, Shunde, Guangdong China; 7 Department of Liver Surgery, Cancer Center, Institute of Precision Medicine, the First Affiliated Hospital of Sun Yat-Sen University, No. 58, Zhongshan 2nd Road, Guangzhou, 510080 Guangdong China

**Keywords:** Deep learning, HCC, Histological, MVI, Multicenter, Multiple instance learning, Neural network, Prediction, Surgical margin, Weakly supervised learning, Whole slide image

## Abstract

**Background:**

Microvascular invasion (MVI) is essential for the management of hepatocellular carcinoma (HCC). However, MVI is hard to evaluate in patients without sufficient peri-tumoral tissue samples, which account for over a half of HCC patients.

**Methods:**

We established an MVI deep-learning (MVI-DL) model with a weakly supervised multiple-instance learning framework, to evaluate MVI status using only tumor tissues from the histological whole slide images (WSIs). A total of 350 HCC patients (2917 WSIs) from the First Affiliated Hospital of Sun Yat-sen University (FAHSYSU cohort) were divided into a training and test set. One hundred and twenty patients (504 WSIs) from Dongguan People’s Hospital and Shunde Hospital of Southern Medical University (DG-SD cohort) formed an external test set. Unsupervised clustering and class activation mapping were applied to visualize the key histological features.

**Results:**

In the FAHSYSU and DG-SD test set, the MVI-DL model achieved an AUC of 0.904 (95% CI 0.888–0.920) and 0.871 (95% CI 0.837–0.905), respectively. Visualization results showed that macrotrabecular architecture with rich blood sinus, rich tumor stroma and high intratumor heterogeneity were identified as the key features associated with MVI ( +), whereas severe immune infiltration and highly differentiated tumor cells were associated with MVI (−). In the simulation of patients with only one WSI or biopsies only, the AUC of the MVI-DL model reached 0.875 (95% CI 0.855–0.895) and 0.879 (95% CI 0.853–0.906), respectively.

**Conclusion:**

The effective, interpretable MVI-DL model has potential as an important tool with practical clinical applicability in evaluating MVI status from the tumor areas on the histological slides.

**Graphical abstract:**

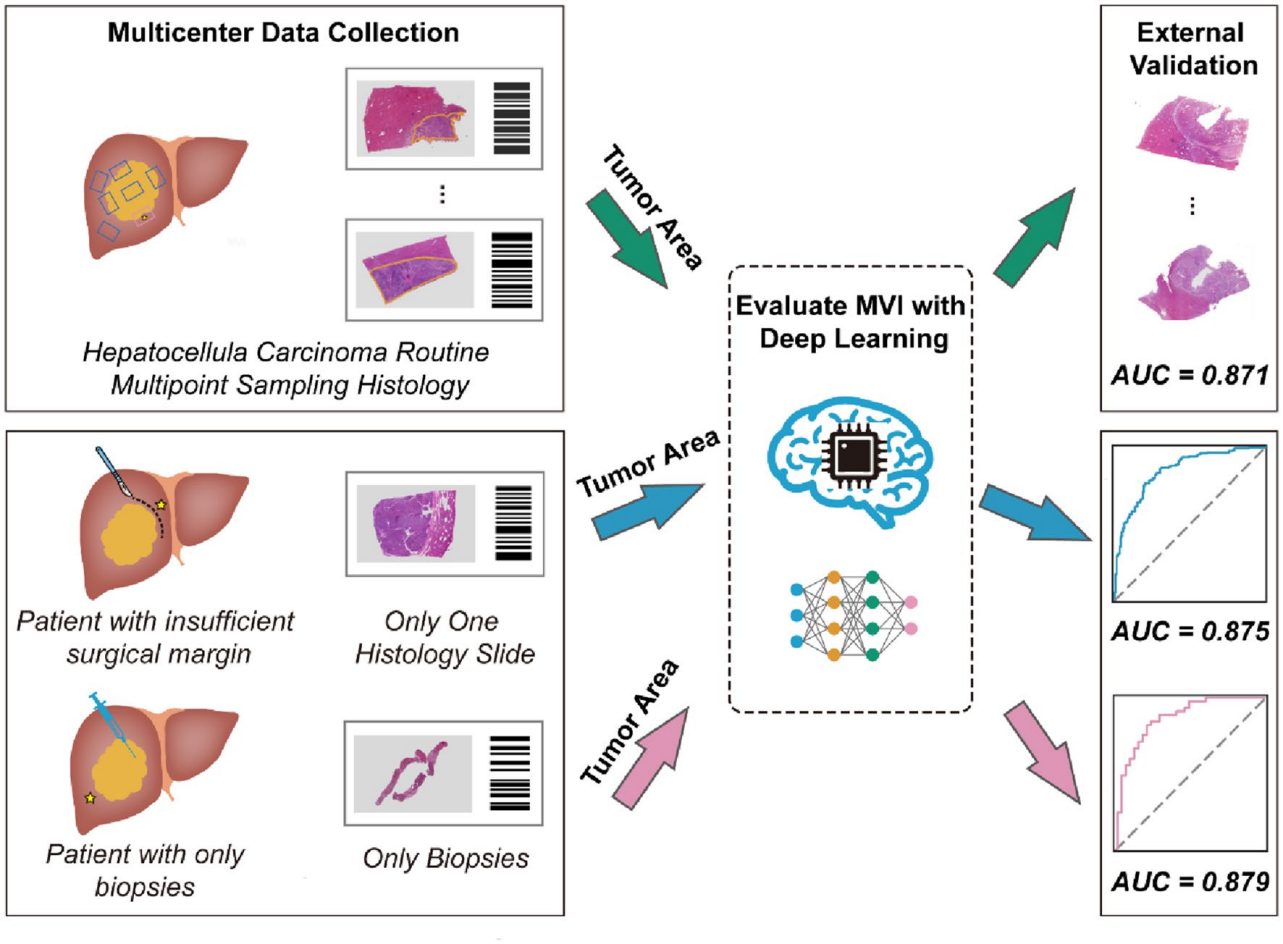

**Supplementary Information:**

The online version contains supplementary material available at 10.1007/s12072-022-10323-w.

## Introduction

Microvascular invasion (MVI) is one of the most important histological features of the prognosis and treatment management of hepatocellular carcinoma (HCC) [1, 2]. Postoperative adjuvant transarterial chemoembolization for HCC patients with MVI significantly reduced tumor recurrence and improved survival [3, 4]. MVI is also the only histological feature that has been proven to be predictive of the efficacy of adjuvant therapy by a clinical trial [5]. Additionally, MVI status can also be an indicator for therapeutic decision-making in recurrent HCC [6]. Therefore, an accurate histological diagnosis of MVI is highly critical to the precise management of HCC.

Postoperative histological assessment is the gold standard for the diagnosis of MVI. However, MVI is commonly scattered in the adjacent peri-tumor liver tissues, leading to difficulties in its evaluation. Accordingly, 83.3% of the MVIs were located within 1 cm from the tumor boundary, but also approximately 8.4% were located beyond 2 cm or even further [7]. Therefore, multipoint sampling at the peri-tumor region is necessary to ensure the detection rate of MVI [8, 9]. Nevertheless, in clinical practice, not all patients could obtain sufficient sampling tissues for MVI evaluation. More than 60% of HCC patients received nonsurgical treatment with only biopsy specimens [10]. For patients who receive surgical treatment, the background cirrhosis leads to a considerable portion of them having narrow surgical margins to maintain sufficient remnant liver volume [11]. According to the previous studies, the proportion of HCC patients with margins < 0.5 cm ranged from 43.6 to 44.2% [7]. The MVI status could hardly be evaluated in these patients with limited information on the peri-tumor region, ultimately affecting the treatment decisions and clinical outcomes.

Histological whole slide images (WSIs) of HCC contain a massive amount of biological information. Recent studies demonstrated that some histological features such as macrotrabecular-massive type, cholangiocarcinoma-like and stem cell-like traits were positively correlated with the incidence of vascular invasion [12, 13]. Therefore, quantitative analysis of this information in tumor tissue has the potential to help diagnose MVI without sampling from the peri-tumor region. However, quantitative evaluation of this information is challenging with the naked eyes of pathologists. Deep learning could automatically extract imaging features which are invisible to human observers and potentially provide important clinical, biological and molecular-morphologic information [14]. Previous studies on deep-learning models in other tumors also indicated the possibility of predicting features outside the tumor (such as lymph node metastasis) by evaluating information from tumor areas only [15, 16]. Therefore, constructing a deep-learning model based on the imaging information of tumor areas might have the potential to evaluate MVI status effectively and assist with the clinical management of HCC patients.

In this study, we developed MVI deep-learning (MVI-DL) prediction model in multicenter HCC cohorts, by learning the characteristic information from the tumor areas of histological WSIs, to automatically and accurately evaluate the MVI status.

## Materials and methods

### Patient cohorts and data preparation

This study protocol conforms to the ethical guidelines of the 1975 Declaration of Helsinki as reflected in a priori approval by the institution's Human Research Committee. Informed consent was waived since this was a retrospective cohort study.

**Fig. 1:**
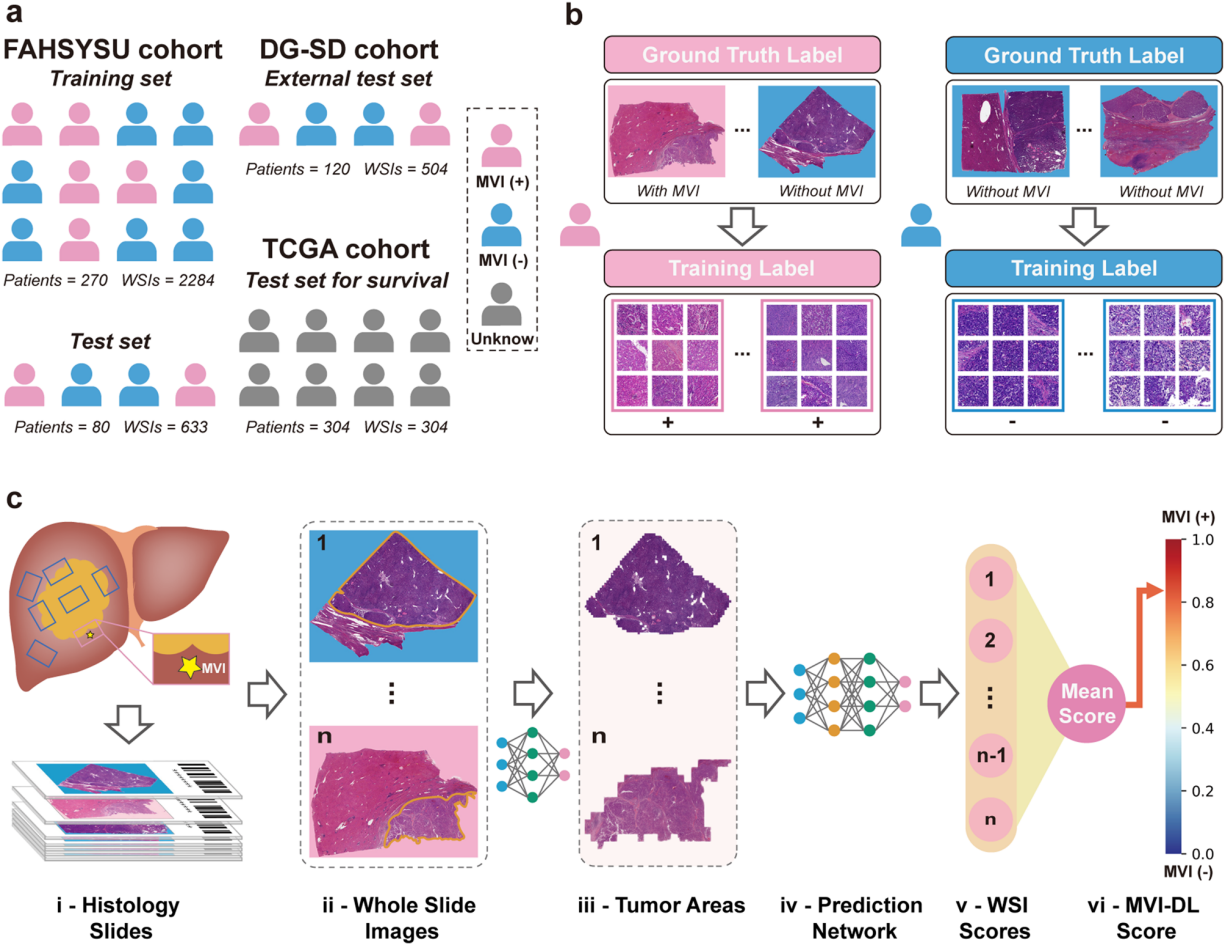
Data collection and study design. **a** Patients from three medical centers and the TCGA dataset were enrolled in this study. **b** Labelling of the images. All patches on one WSI were considered as a patch bag and shared a same label. If the patient is MVI (−), all its WSIs, namely, patch bags would be labelled as negative; If the patient is MVI ( +), all the patch bags would be labelled as positive, regardless of the existence of MVI. **c** The flowchart of the MVI-DL model. All WSIs obtained from multipoint sampling were automatically segmented first, and the tumor areas tiled at different magnification scales were then fed into the prediction network. The average of all WSI-level scores formed the MVI-DL score of the patient, and when it is above a certain threshold, the patient is predicted to be MVI ( +)

We retrospectively collected 368 patients from the First Affiliated Hospital of Sun Yat-sen University (FAHSYSU) and 120 patients from the Dongguan People’s Hospital and Shunde Hospital of Southern Medical University (DG-SD) who underwent curative hepatectomy from January 2016 to December 2018. All these patients were pathologically diagnosed with HCC, and all hematoxylin and eosin histological slides were collected to train and validate the MVI prediction model. Slides with poor staining quality or images with artifacts after scanning were excluded. Finally, a total of 2917 WSIs of 350 patients from FAHSYSU were included and were randomly divided into a training set and an independent test set. A total of 504 WSIs of 120 HCC patients from DG-SD formed an external test set (Fig. 1a). In order to ensure the high-quality ground truth labels of the data used for model development and validation, all the resected HCC specimens from the FAHSYSU and DG-SD cohorts had surgical margins over 2 cm, and sufficient postoperative sampling tissues were obtained for MVI evaluation. According to our previous large retrospective study, a threshold of four, six, eight and eight sampling tissues within peri-tumor liver parenchyma were required for evaluating MVI in solitary tumors measuring 1.0–3.0 cm, 3.1–4.9 cm and ≥ 5.0 cm and multiple tumors [8]. Additionally, the histological diagnosis of MVI for each slide was prospectively evaluated based on the consensus of three pathologists with over 5 years of experience in liver pathology.

Additionally, a total of 376 WSIs from 376 HCC patients were obtained from the TCGA database via the Genomic Data Commons (https://gdc.cancer.gov/). Information on recurrence-free survival (RFS) and overall survival (OS) was collected from these patients. Patients without survival data or slides with poor image quality were excluded and finally 304 WSIs of 304 HCC patients were included to evaluate the correlation of predicted MVI results by the MVI-DL model with patients’ survival outcomes (Fig. 1a).

All WSIs were scanned at 40 × magnification by a KF-PRO-020 type of scanning machine (KFBIO, Ningbo, China) and were stored in SVS file format. A pathologist with over 1 year of working experience in liver pathology performed the image quality control to screen out poorly stained slides or had obvious artifacts. To prevent overlaps between datasets, the WSIs from a given patient were kept together in the same set. To extract the information under different magnifications, we divided the WSIs into nonoverlapping 512 × 512 pixel patches at magnifications of 5 × , 10 × , 20 × and 40 × , respectively. Patches with over 50% of background coverage were excluded (Supplementary Methods).

### Development of the MVI-DL model

#### Image sampling and magnification selection

To clarify the contribution of different tissue areas in the WSI to the prediction of MVI, and to further confirm the sampling strategy for model training, we compared the performances of models developed based on different tissue areas. We first trained a segmentation network, details in Supplementary Methods. We then applied the segmentation model to all other WSIs in the FAHSYSU and DG-SD cohorts for automatic segmentation. Prediction models were constructed based on tumor area, peri-tumor area and the whole WSI, respectively, to compare models’ performance with different sampling strategies.

To further determine the optimal number of sampling patches under different magnifications for the prediction model, we performed sensitivity analyses of the number of sampling patches under different magnifications. We also compared the performances of the models under different magnifications with the ensemble model integrating different magnifications to determine the network structure of the final prediction model (MVI-DL model).

#### Training of the MVI-DL model

**Fig. 2:**
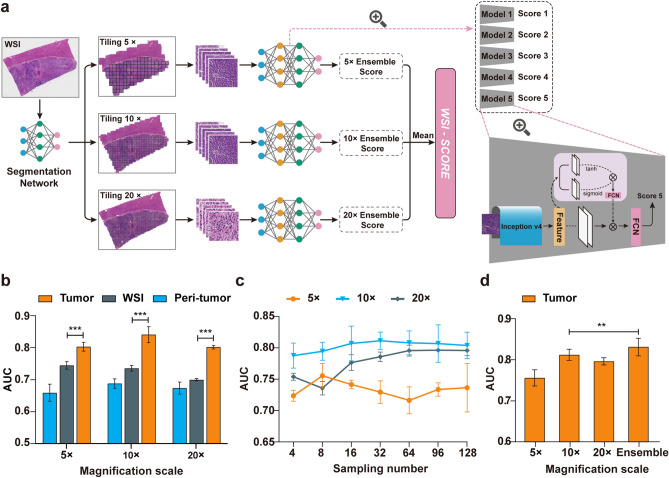
Network structure and hyperparameters of the MVI-DL model. **a** The MVI-DL model consisted of a segmentation model and a predication model. First, the segmentation model identified the tiled patches as tumor or peri-tumor patch under 5 × , 10 × and 20 × magnification scales. The prediction section included five trained models (each model consisted of an Inception-v4 network, a MIL pooling layer and a fully connected layer) at each magnification scale, these tumor patches were fed into the five models and each generated one score reflecting the probability of MVI. The average of the five scores was considered as the ensemble score, one for each magnification. The mean of the three ensemble scores represents the final predictive score for this WSI. **b** Comparison of the AUCs of different sampling tissue categories under different magnification scales. **c** Comparison of the AUCs of different sampling patches numbers under different magnification scales. **d** Comparison of the AUCs of single magnification scales and the ensemble one. ns, *p* > 0.05; *, 0.05 > *p* > 0.01; **, 0.01 > *p* > 0.001; ***, *p* < 0.001

The MVI-DL model was constructed based on a weakly supervised multiple-instance learning (MIL) framework [17]. The framework consists of a convolutional neural network (CNN) feature extraction layer, a MIL pooling layer and a fully connected layer. Each WSI obtained a patch bag after tiling, the label of which was the patient’s MVI status (Fig. 1b). We used patch bags and their corresponding labels as the input to train the prediction network. A pre-trained Inception-v4 model was used as the backbone to extract the features of patches. In the MIL pooling layer, we introduced the attention mechanism, aggregated the patch features through the attention score, and finally output the predicted value of the WSI through the fully connected layer (Fig. 1c and Supplementary Methods). We used a fine-tuned set (a part of the training set) to select five optimal prediction models before overfitting and took the average of the five models as the final prediction score (Fig. 2a and Fig. S1, S2). The average of the prediction scores under different magnifications formed the prediction scores of the MVI-DL model.

#### Visualization of the MVI prediction

To further understand the key histological features that contribute the most to the model prediction of MVI, we extracted the top 4000 and the bottom 4000 patches based on the MVI predictive attention score and then clustered and visualized them using t-SNE and DCCS algorithms [18, 19]. Pathologists reviewed the pathological features in each cluster of patches without being informed of the label or prediction score for each patch. We also applied gradient-weighted class activation mapping (Grad-CAM) [20] to provide an insight into regions within each patch of the corresponding cluster that the MVI-DL model used to generate predictions (Supplementary Methods).

### Validation of the MVI-DL model performance

The predictive performance of the MVI-DL model was evaluated in the FAHSYSU test cohort and DG-SD cohort. Clinical information (age, gender, serum AFP level, tumor number and size, BCLC, Edmondson grade and tumor encapsulation) was collected and analysed by multivariable logistic analysis to determine the MVI-associated clinical characteristics. A clinical-MVI-DL model was constructed based on the MVI-DL prediction score and the clinical MVI-associated characteristics. We further compared the performance of the MVI-DL model with MVI-associated clinical characteristics and the clinical-MVI-DL model.

For patients in the FAHSYSU test cohort, the DG-SD cohort and the TCGA cohort, we further divided them by the predicted MVI status from the MVI-DL model. Subsequently, we compared the RFS and OS between patients predicted to be MVI ( +) and MVI (−) to evaluate the correlation between the predicted MVI status and patient’s survival outcomes in these three cohorts independently.

### Simulation of the clinical application of the MVI-DL model

#### Patients with insufficient surgical margin for MVI evaluation

Considering that some HCC patients with narrow surgical margins had insufficient histological sections for MVI evaluation, we simulated a clinical scenario where patients had only one WSI. We randomly selected one WSI for each patient in the FAHSYSU and DG-SD test set as the input and then analysed the predictive performance of the MVI-DL model. Additionally, to further investigate the impact of different number of the WSIs randomly selected from each patient on the prediction performance of the model, we performed 100 rounds of iteration for each point of the number of the WSIs and then performed a sensitivity analysis with these mean values (standard deviation) of each point that calculated from the generated 100 results.

#### Patients with biopsies only

Considering that a large part of HCC patients could only acquire biopsy specimens, we simulated a clinical scenario where the tissue size of the WSIs were similar to liver biopsy specimens. Therefore, we randomly selected one WSI for each patient in the FAHSYSU and DG-SD test set and then randomly sampled adjacent patches with a similar area of a liver biopsy from each WSI as the one simulated biopsy. Clinically, three biopsies at most were routinely acquired for HCC patients. Therefore, we analysed the predictive performance of the MVI-DL model with one to three simulated biopsies. The detailed simulation method is described in Supplementary Methods.

### Statistical analysis

Receiver operating characteristic (ROC) curves were generated to evaluate the performance of the MVI-DL model. Subsequently, the area under the receiver operating characteristic curve (AUC) values were calculated accordingly. A two-sided DeLong test was used to compare the AUCs. An optimal cut-off was determined by the ROC curve to reach the best accuracy, which was 0.58 in this study. The accuracy, sensitivity and specificity were then calculated according to this cut-off for the prediction results (≥ 0.58 as positive, < 0.58 as negative). RFS and OS curves were analysed using the Kaplan–Meier method and compared using the Mantel–Cox log-rank test. Logistic regression analyses were performed to select the MVI-associated characteristics. Each variable was assessed by univariate logistic regression analysis, and variables with a *p* < 0.05 were enrolled in a stepwise multivariate analysis. The clinical-MVI-DL model was then constructed based on the results of multivariable logistic regression analysis. A two-sided *p* value less than 0.05 was considered statistically significant. Scikit-learn was used for ROC curve analysis and the calculation of the confusion matrix.

## Results

### Patient characteristics

We initially obtained 3568 slides from 488 HCC patients across two independent cohorts. A total of 147 (4.1%) slides from 22 patients who did not meet the inclusion criteria were excluded. Finally, in the FAHSYSU cohort, a total of 2917 slides from 350 HCC patients were enrolled, 180 (51.4%) of whom were MVI ( +), while in the DG-SD cohort, 504 slides from 120 HCC patients were enrolled, 44 (36.7%) of whom were MVI ( +). Patients from the FAHSYSU cohort were randomly divided into a training set and an independent test set. Patients from the DG-SD cohort were used as the external test set (Fig. 1a). Baseline clinical and demographic characteristics were generally well balanced between the two cohorts, except for a significantly higher Edmondson grade (*p* = 0.009) and higher incidence of MVI ( +) (*p* = 0.015) in the FAHSYSU cohort (Table S1).

### Development of the MVI-DL model

Considering that there is a huge amount of information on a single WSI, we first trained a tissue segmentation network to automatically segment all WSIs in the two cohorts (Table S2 and S3) and compared the contribution of different tissue areas on the WSI to MVI prediction to reduce the redundant information. One WSI was divided into the tumor area and peri-tumoral area by a segmentation network (Fig. S3). We tested the performance of our segmentation network, achieving accuracies of 0.960, 0.958, 0.934 and 0.932, and AUCs of 0.991, 0.986, 0.984 and 0.981, under 5 × , 10 × , 20 × and 40 × magnification scales, respectively (Fig. S3).

Then, we used patches from the tumor area, peri-tumoral area and the whole WSI as inputs to construct prediction models for MVI and compared the performances between different tissue categories and different magnification scales. The predictive performances of the models under 40 × magnification were significantly lower than those of other magnifications, with the best AUC of only 0.68, and were excluded from the following analysis. The models using patches from the tumor area as the inputs had significantly higher AUCs than those using patches from the peri-tumoral area or the whole WSI at every magnification scale (all *p* < 0.001, Fig. 2b).

Next, we evaluated the optimate sample counts for the input patches and select the sample count with the best training efficacy under each magnification scales. We chose 8 patches for the 5 × , 32 patches for the 10 × and 64 patches for the 20 × magnification scales to achieve the best training efficacy and sufficient sampling numbers to represent the features of the tumor area (Fig. 2c). The ensemble model integrating different magnification scales reached an AUC of 0.831 for tumor area and was significantly higher than those under a single magnification scale (*p* = 0.004, Fig. 2d). Therefore, we took the ensemble model for the tumor area as the backbone model for the following training and validation of the MVI-DL model (Fig. 2a).

### Validation of the MVI-DL model

**Fig. 3:**
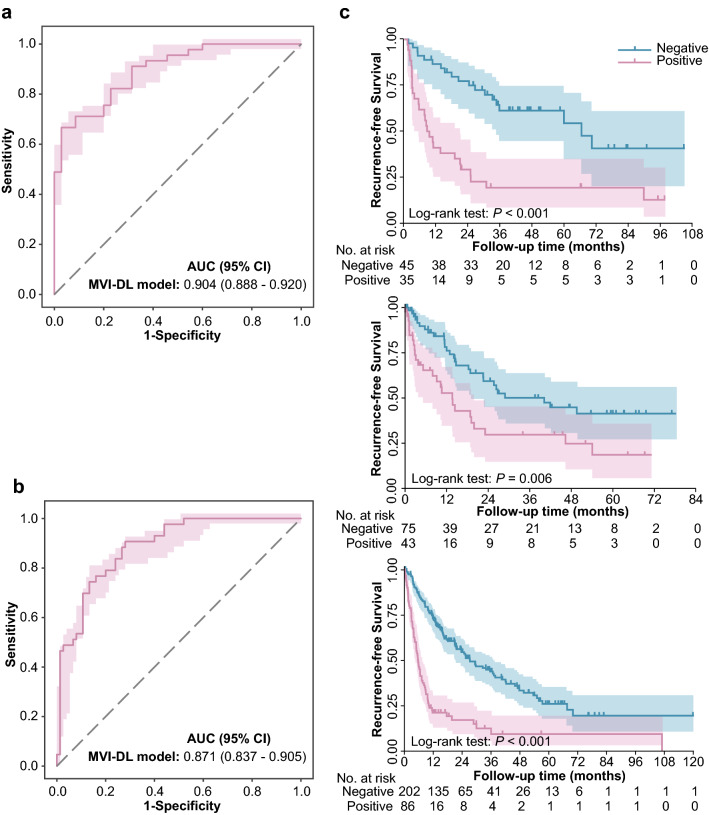
Performances of the MVI-DL model on the test sets. The AUCs evaluated on the FAHSYSU (**a**) and DG-SD (**b**) test set. **c** Kaplan–Meier curves for RFS analysis of the patients stratified by the MVI-DL model in the FAHSYSU (top), DG-SD (middle) and TCGA (bottom) test sets. RFS, recurrence-free survival

We validated the performances of the MVI-DL model on the FAHSYSU and DG-SD test set (Fig. 3a, b). On the FAHSYSU test set, the AUC of the MVI-DL model was 0.904 (95% CI 0.888–0.920), and the accuracy, sensitivity and specificity were 83.3%, 92.6% and 71.0%, respectively. For the DG-SD cohort, the AUC reached 0.871 (95% CI 0.837–0.905), and its accuracy, sensitivity and specificity were 79.1%, 90.0% and 69.8%, respectively (Table S4).

Furthermore, we also compared the RFS and OS between patients predicted with different MVI status, and the results showed that patients who were predicted to be MVI ( +) had significantly worse survival outcomes than patients with MVI (−) (median RFS: 9.39 vs. 66.15 months, *p* < 0.001; 13.09 vs. 40.20 months, *p* < 0.001; 7.23 vs. 36.70 months, *p* < 0.001 in the FAHSYSU test set, DG-SD cohort and TCGA cohort, respectively) (Fig. 3c). The OS analysis also indicated the similar results (Fig. S4).

To compare the predictive performance of the MVI-DL model to the clinical characteristics and the combined clinical-MVI-DL model, we performed a univariable and multivariable logistic regression analysis of factors associated with MVI in the training set (Table S5). Multivariable analysis revealed that the MVI-DL prediction score (OR 1.07, 95% CI 1.05–1.09, *p* < 0.0001) was an independent predictive factor of MVI and was higher than the combined clinical score (*p* < 0.001, Fig. S5). The combination of the clinical score with the MVI-DL model did not improve the predictive performance of the MVI-DL model (*p* = 0.304 and 0.289 for the FAHSYSU and DG-SD test set, respectively).

### Visualization and interpretability of the MVI-DL model

**Fig. 4:**
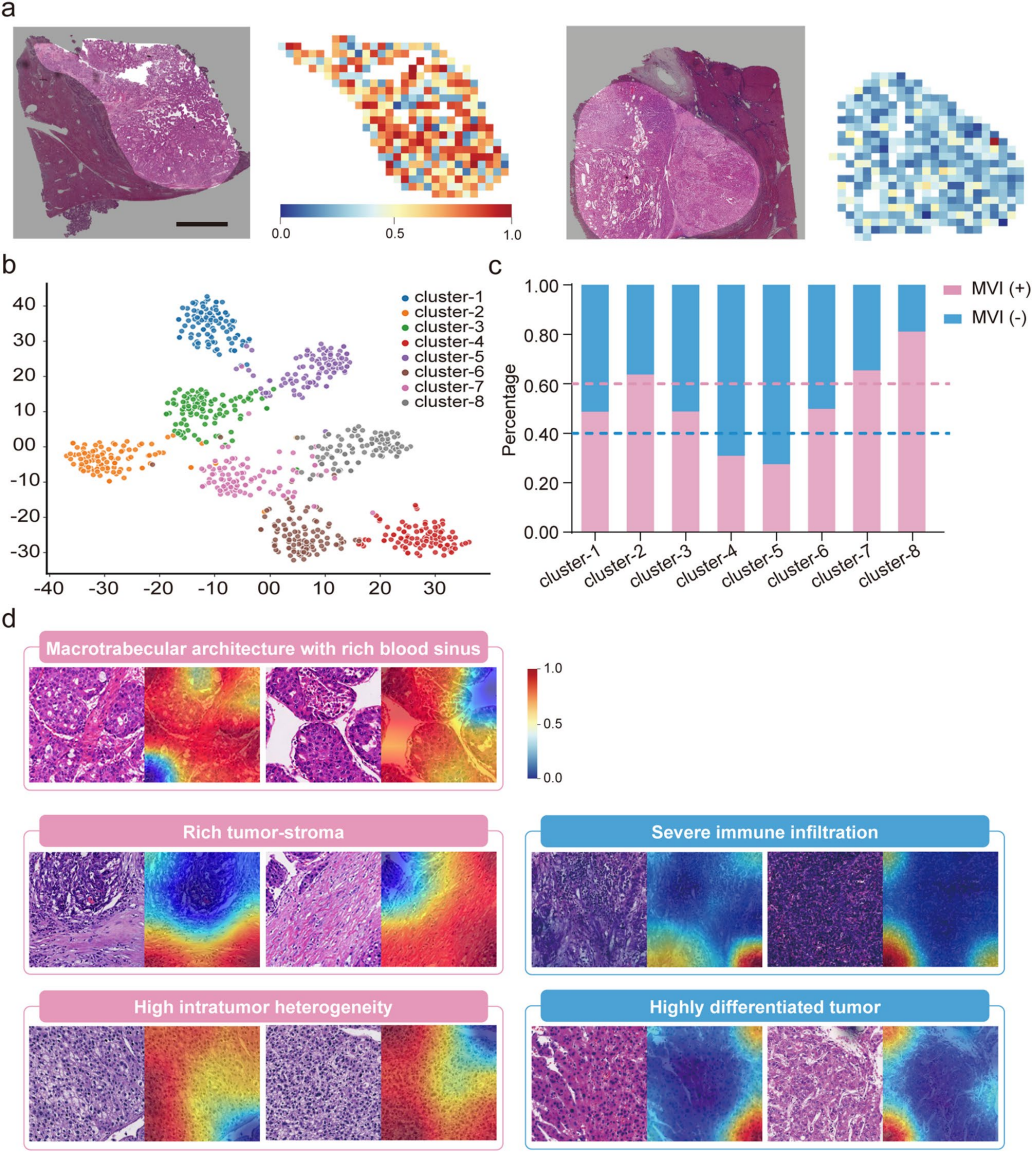
Visualization and clustering of the risk heatmaps. **a** One example of heatmaps of the WSIs predicted with MVI ( +) and MVI (−) by the MVI-DL model. Original WSIs (left); Heatmaps (right). **b** Unsupervised cluster analysis of the top 4000 and the bottom 4000 patches based on the attention score by t-SNE and DCCS algorithms. **c** Proportion of the patches predicted as MVI ( +) or MVI (−) in the eight clusters; Pink dotted line represents the proportion of the patches predicted as MVI ( +) in this cluster exceeded 60%; Blue dotted line represents the proportion of the patches predicted as MVI (−) in this cluster exceeded 60%. **d** Visualization of represented patches and their corresponding grad-CAM in the predictive clusters. The colour scheme represents the calculated weight of probability at each region, which indicates the contribution of the corresponding area to the model prediction (red area indicates the region with most important contribution, while blue area indicates less contribution)

Figure 4a shows two WSIs predicted with MVI ( +) and MVI (−) by the model and the corresponding heatmaps as examples. The unsupervised classification classified the patches into 8 clusters (Fig. 4b). Clusters with over 60% patches from MVI ( +) patients were defined as MVI ( +)-related clusters, while those with over 60% patches from MVI (−) were defined as MVI (−)-related clusters (Fig. 4c). We found that high intratumor heterogeneity (Cluster 2), rich tumor stroma (Cluster 7), and macrotrabecular architecture with a rich blood sinus (Cluster 8) were associated with MVI ( +). In contrary, severe immune infiltration (Cluster 4) and highly differentiated tumor cells (Cluster 5) were associated with MVI (−) (Fig. S6). Grad-CAMs also showed that regions occupied by these features received higher or lower weights (Fig. 4d).

### Clinical implementation of the MVI-DL model

**Fig. 5:**
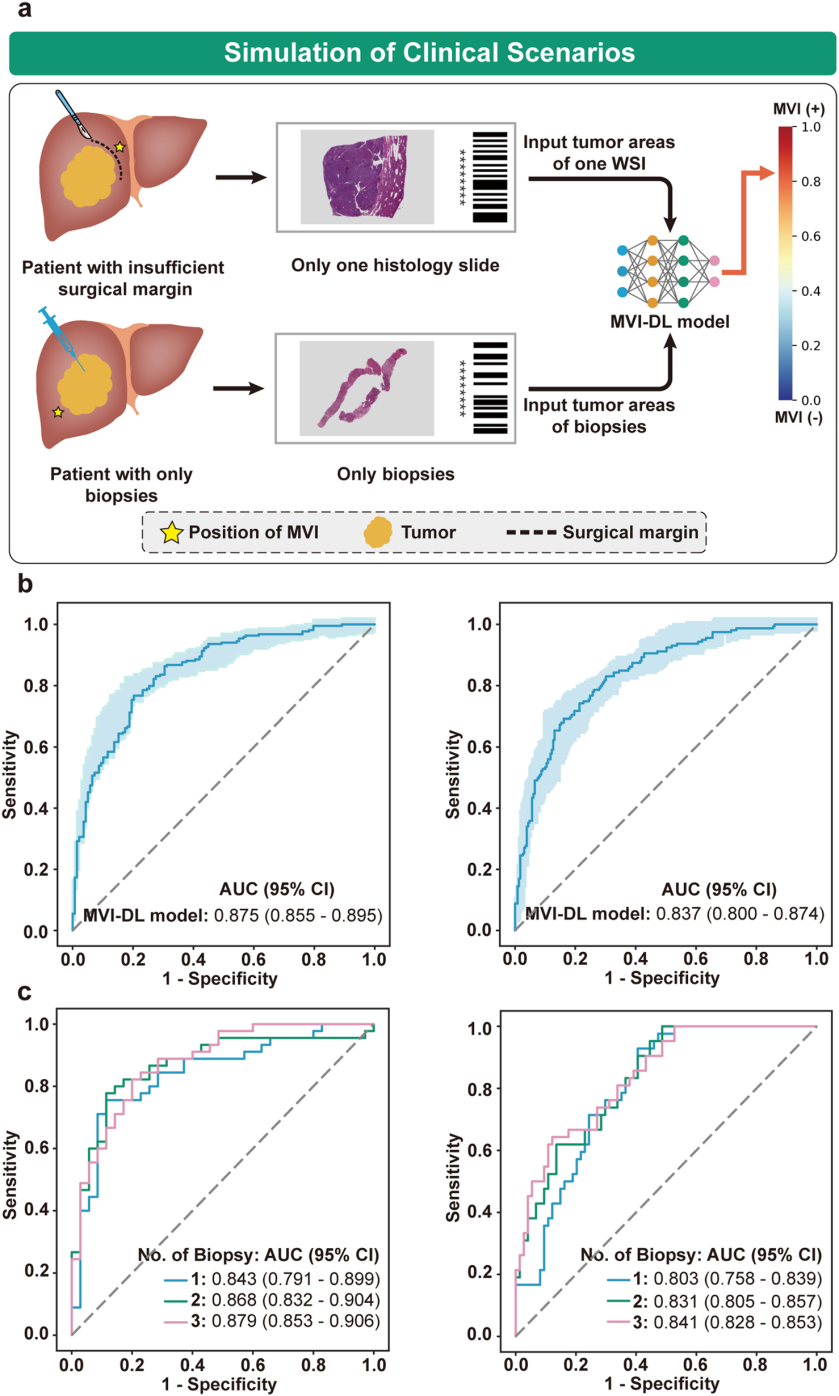
Simulation of MVI-DL model in clinical scenarios. **a** We simulated the clinical scenarios where patient with an insufficient surgical margin had only one WSI and patient with only biopsies. The MVI-DL model used one WSI or biopsies as the input and output an MVI-DL score for those patients who used to be not able to be evaluated. **b** The AUC of the MVI-DL model predicted with only one WSI in the FAHSYSU (left) and DG-SD (right) test set. **c** The AUCs of the MVI-DL model predicted with different biopsy number in the FAHSYSU (left) and DG-SD (right) test set

Clinically, patients with limited surgical margins or patients who underwent ablation therapy could hardly acquire sufficient histological sections to evaluate MVI. Therefore, we next simulated the implementation of the MVI-DL model in these two clinical scenarios (Fig. 5a). First, we validated the performance of the MVI-DL model using only one WSI from each patient in the FAHSYSU and DG-SD test set to simulate patients with limited surgical margins. The results showed that the AUC was 0.875 (95% CI 0.855–0.895) and 0.837 (95% CI 0.800–0.874), respectively (Fig. 5b). The detailed evaluation metrics are shown in Table S6. Additionally, we also performed a sensitivity analysis to explore the impact of the number of the WSIs per patient fed to the model on the performance of the MVI-DL model, and the results showed that the predictive accuracy of the model did not increase significantly as the number of the input WSIs increased (the AUCs range from 0.849 to 0.878, Fig. S7). Then, we validated the performance of MVI-DL model in simulated biopsy specimens to evaluate the performance of the MVI-DL model in patients with only biopsy tissues. The results showed that the predictive performance positively correlated with the number and length of simulated biopsy tissues (Fig. 5c, Table S6 and Fig. S8), and the AUC reached 0.879 (95% CI 0.853–0.906) by three biopsies and similar trends were shown in the external test set.

## Discussion

In this study, we constructed MVI-DL model for MVI evaluation in HCC patients. The MVI-DL model was well-validated in the independent external cohort. We found that the tumor areas contributed the most to the MVI-DL model, indicating that some imaging features of the tumor area were strongly associated with the existence of peri-tumoral MVI. We then identified these histological features by clustering and visualization. We further simulated the clinical scenarios where tissue samples were insufficient for MVI evaluation, and the results showed that the MVI-DL model could accurately diagnose MVI in these scenarios.

The precise histological diagnosis of MVI is critical for managing HCC patients but faces two main challenges in clinical circumstances. On the one hand, multipoint sampling of peri-tumor is essential for accurate diagnosis of MVI [8, 9] but multiplies the diagnosis time of pathologists. On the other hand, a considerable proportion of patients had insufficient peri-tumor regions to evaluate MVI status [10]. To solve these clinical dilemmas, we first retrospectively collected all the WSIs of those HCC patients from two cohorts with high-quality MVI labels, and we then established an MVI-DL model for MVI evaluation with high accuracy and was well validated in external cohorts. By automatically analysing WSIs, the MVI-DL model can reduce the workload of pathologists in MVI evaluation greatly. Furthermore, we verified the model in two simulated clinical scenarios, where the patient had only one single section or only a biopsy specimen and achieved similar performances. Additionally, we also confirmed that the overall predictive accuracy did not increase significantly as the number of the input WSIs increased. These results not only indicated the possibility of predicting MVI status in patients who could not be evaluated before, but also greatly reduce the increased workload caused by multipoint tissue sampling. By applying the MVI-DL model, a pretreatment biopsy could reflect the MVI status and further contribute to various therapeutic decision-making. Physicians could plan to achieve a surgical margin > 1 cm for MVI ( +) patients to reduce postoperative recurrence [7], design safe ablation margins for RFA in MVI ( +) patients [21], and rearrange the priority of the liver transplantation list [22].

Although MVIs are located in the peri-tumoral area, our results showed that the tumor area-based model had the best performance. The scattered distribution of MVI could cause difficulties in constructing a detection model directly from the peri-tumoral area. Not all slides from MVI ( +) patients contain MVIs leads to a high false-positive rate in MVI labels. The tumor area-based MVI-DL model we constructed predicts the existence of MVI by certain MVI-associated histological features in the tumor area instead of trying to find the location of MVI, avoiding the inconsistent labels in different slides. Interestingly, we found that the performances of tumor area-based models were diverse under different magnifications. The 40 × model showed limited predictive efficacy compared with the lower magnifications. The reason could be that images under different magnifications reveal different types of histological features [23]. Images under 40 × magnification better reflect the characteristics of tumor cell morphology and internal cell structure, while images under lower magnifications reflect the tumor cell morphology and the relationship between tumor cells and their surrounding microenvironments, such as tumor-associated stromal cells and tumor-infiltrated lymphocytes. Therefore, we speculate that the relationship of tumor cells and their surrounding microenvironments may be more important for predicting MVI. By assembling models under different magnifications, the final MVI-DL model better combined cell morphology, tumor microenvironment and intercell relationships, and achieved better predictive performance.

This is confirmed by the unsupervised clusters and heatmaps of our study. We found that macrotrabecular architecture with a rich blood sinus, rich tumor stroma, high intratumor heterogeneity, severe immune cell infiltration and highly differentiated tumor cells strongly contributed to the MVI prediction, all of which were features of cell morphology, tumor microenvironment and intercell relationships. Previous studies have shown that macrotrabecular architecture in HCC indicates stronger tumor invasiveness [12, 24]. This type of HCC can express high levels of angiopoietin 2 and vascular endothelial growth factor A to regulate angiogenesis and vascular remodeling and is more likely to develop vascular invasion and metastasis [12, 25]. A rich tumor stroma may promote the production of TGF-β, which directly upregulates the expression of tumor stem cell markers (EpCAM, K19, CD133, etc.), thereby promoting vascular invasion [13]. High intratumoral heterogeneity may also be related to stronger tumor invasiveness. Studies have revealed that high intratumoral heterogeneity affects key cancer pathways and drives phenotypic variation [26], and ultimately promotes tumor progression and metastasis through a complex intercell competition mechanism [27]. In contrast, highly differentiated tumor cells and immune cell infiltration are closely related to the reduction of postoperative tumor recurrence and better prognosis [28–30]. Tumor immune cell infiltration has also been proven to be negatively correlated with vascular invasion in colorectal cancer [31]. These results indicated high interpretability and clinical reliability of the MVI-DL model, which are essential for clinical acceptability.

There are still some limitations in this study. First, the MVI-DL model was constructed and validated in three medical centers, all of which were from China. Therefore, population of this study was populated mainly with HBV-related HCC patients. The generalizability of this model to HCC with other etiologies needs further validation. Second, the performance of this model in biopsy specimens was in a simulated scenario. Since that the diagnosis of HCC did not require preoperative biopsy, patients with both preoperative biopsy tissue and confirmed MVI diagnosis from postoperative histology were limited, making it hard to evaluate in this population. Third, this study is a retrospective cohort study, and a large prospective clinical trial is necessary for the implementation of the MVI-DL model in clinical practice.

## Conclusions

The efforts presented in our work highlighted the possibility of accurately evaluating the MVI status of HCC patients from the tumor area on the histological slides using a deep-learning model. With the validations on multicenter cohort, the MVI-DL model we developed exhibited excellent accuracy, robustness and considerable clinical interpretability, which might provide an important tool with practical clinical applicability for better patient management.

## Supplementary Information

Below is the link to the electronic supplementary material.

Supplementary file1 (DOCX 53 KB)

Supplementary file2 (TIF 685 KB)

Supplementary file3 (EPS 1018 KB)

Supplementary file4 (TIF 3592 KB)

Supplementary file5 (EPS 1216 KB)

Supplementary file6 (EPS 959 KB)

Supplementary file7 (TIF 8307 KB)

Supplementary file8 (EPS 1289 KB)

Supplementary file9 (EPS 887 KB)

## Data Availability

The external validation of TCGA dataset is publicly available at the TCGA portal (https://portal.gdc.cancer.gov). All other data generated in this study are not publicly available due to patient privacy constraints, but are available upon reasonable request from the corresponding author (Kuang).

## Code availability

The codes that were used to train and validate the deep-learning model in the manuscript were available from the corresponding author (Kuang) upon reasonable request.
