# Supplementary material for: Deep learning for evaluation of microvascular invasion in hepatocellular carcinoma from tumor areas of histology images
Source: Hepatol Int. 2022 Mar 28;16(3):590–602. doi: 10.1007/s12072-022-10323-w (PMC9174315; doi:10.1007/s12072-022-10323-w)
Supplement: Supplementary file 1 — Supplementary file1 (DOCX 53 KB) [file 12072_2022_10323_MOESM1_ESM.docx]

**Deep Learning for Evaluation of Microvascular Invasion in Hepatocellular** **Carcinoma from Tumor Areas of Histology Images**

**Table of contents**

[**Supplementary Methods**](#_Toc3951)

**Supplementary References**

**Table S1** Baseline characteristics of the patients in the FAHSYSU and DG-SD cohort

**Table S2** Data used to train the segmentation network

**Table S3** Data used to train and test the MVI-DL model

**Table S4** Predictive performance of the MVI-DL model evaluated on the FAHSYSU and DG-SD test set

**Table S5** Univariate and multivariate analysis of factors associated with MVI in the training set

**Table S6** Performance of the MVI-DL model in application to two simulated clinical scenarios

[**Supplementary**](#_Toc3951) **Figure Legends**

[**Supplementary Methods**](#_Toc3951)

**Data Preprocessing and Augment**

All WSIs were tiled into multiple nonoverlapping 512×512 pixel patches at 5×, 10×, 20× and 40× magnification scales using the Python interface (version 1.1.2) of Openslide-3.4.1 (https://openslide.org/). Background coverage was calculated with the threshold of 225 after adjusting the brightness and contrast of the patch through Pillow 6.1.0.

In the segmentation and prediction tasks, patches were standardized by the mean and variance calculated from the overall dataset. During the training, each patch was randomly cropped to 384×384 pixel and then resized to 299×299 pixel; Additionally, we performed random horizontal flipping and color jitter on patches. While in the validation, only center crop and resize were implemented to maintain the same input size. All data augmentation methods were implemented by Torchvision-0.8.1.

**Segmentation Network**

In the development of the segmentation network, the tumor and the peri-tumor areas of 974 WSIs in the training set were manually annotated on QuPath-0.2.0 software (open source software; <https://github.com/qupath>) by two pathologists with over 3-year experiences in liver pathology using different colors (red for tumor tissue and green for peri-tumor tissue). Pathologists were required to annotate the areas which were confirmed to be tumor/peri-tumor only. Then a third pathologist with over 5-year experience in liver pathology confirmed the annotation results. The manually annotated results were used as the labels of patches in the corresponding area. The labelled WSIs were allocated at a ratio of 7:3 to train and validate the segmentation model. We trained four different segmentation networks under 5×, 10×, 20× and 40× magnification scales, and then calculated the AUC value to evaluate the performance of these segmentation networks. After training and validating the segmentation model, we applied it to the automatic segmentation of all WSIs in the FAHSYSU and DG-SD cohort.

***Implementation Details:*** We selected the Inception-v4 architecture pretrained on the ImageNet (<https://github.com/Cadene/pretrained-models.pytorch>) and modified the last fully connected layer to reduce the output dimension to 2 (represented two probability values of each patch for the tumor or peri-tumor tissue). We calculated the loss between the predicted probability and the ground truth label corresponding to the patch bag using the cross-entropy loss function, and applied the stochastic gradient descent (SGD) optimizer (weight decay=5e^-4^, momentum=0.95) to update the model parameters. We initialized the learning rate to 1e^-3^ and reduced it by a factor of 0.1 at epoch 15 and 20 (30 training epochs in total).

**Prediction Network**

The MVI-DL model combined a convolutional neural network (CNN) and a MIL framework, consisting of a CNN feature extraction layer, a MIL pooling layer and a fully connected layer. We used patch bags and their corresponding labels as the input to train the prediction network. For the CNN feature extraction layer, we used the feature extractor of the pretrained Inception-v4 (<https://github.com/Cadene/pretrained-models.pytorch>) as the backbone to extract the features of the patches, and the length of the output features was *M*. For the MIL pooling layer, we referred to the attention-based MIL pooling structure [1] and used a weighted average of instances to generate the final representation of the bag. Let $H= \left\{ h_{1},h_{2}\ldots,h_{N} \right\}$ be a bag of feature extracted from CNN Layer, then the aggregated feature of a bag would be defined as follows:

$$Z= \sum_{i=1}^{N} a_{i}h_{i}$$

The weights *a_i_* were determined by a neural network and must sum to 1 to be invariant to the size of a bag. We also introduced the gating mechanism that removes the troublesome linearity in tanh$(\cdot)$ by limiting the final expressiveness among instances.

$$a_{i}= \frac{\exp\left\{ \mathbf{w}^{\top}\left( \tanh\left( \mathbf{V}\mathbf{h}_{i}^{\top} \right)⨀sigm\left( \mathbf{U}\mathbf{h}_{i}^{\top} \right) \right) \right\}}{\sum_{j=1}^{N} \exp\left\{ \mathbf{w}^{\top}\left( \tanh\left( \mathbf{V}\mathbf{h}_{i}^{\top} \right)⨀sigm\left( \mathbf{U}\mathbf{h}_{i}^{\top} \right) \right) \right\}}$$

Where $\mathbf{w}\in\mathbb{R}^{L \times1}$ and $\mathbf{V, U}\in\mathbb{R}^{L \times M}$ are parameters, ⊙is an element-wise multiplication, $\mathrm{sigm}(\cdot)$ is sigmoid function.

***Implementation Details:*** We trained the model by a mini-batch (batch size=8) method and each batch contained *N* patches of a bag randomly sampled from a WSI. During training, we increased the learning rate to the initial set value through a warm-up strategy (epoch = 5), and then adjusted the learning rate through Cosine Annealing. The learning rate was initially set to 1e^-3^ and then successively reduced to 1e^-6^. The logarithmic loss function and the ground truth label were used to calculate the loss value. The SGD optimizer (momentum = 0.9, weight-decay = 5e^-4^) was used to update the model parameters and the accuracy of the model was converged by repeated iteration.

All experiments were run under eight TITAN RTX GPU, the training code was implemented in Torch 1.7.0 framework, parallel training of the model was implemented by NVIDIA/Apex 0.1.0 (opt level was set to O0 and sync batch normalization was open), and the intermediate process was recorded by TensorboardX-2.0. The *Dataloader* was obtained by rewriting *torch.utils.data.distributed.DistributedSampler*, so that the patch bags can be evenly distributed on 8 GPUs for calculation. All random operations involved in the study were implemented by *torch.randperm* and *torch.Generator* methods, and different seeds were used in each experiment.

When validating the 5× model, the sampled value was set to 128, while 512 for the 10× and 20× model. This procedure was repeated 5 times, and the average value was considered as the final value.

**Visualization of MVI-related histopathological features**

***Heatmap:*** The heatmaps were generated over the attention score of the patches. For each WSI, we rescale the attention score, and the function was $ak^{'}= \left( ak-a\_min \right) / \left( a\_max-a\_min \right)$, each grid represents a patch, and the color shade was proportional to the value of $ak'$, namely, the emphasis degree of the model attaches to this patch, and the darker the red, the greater the attention score.

***Cluster Analysis:*** Basing on the statistics of the attention score in the FAHSYSU test set, we clustered the top 4000 and bottom 4000 patches based on the attention score in MIL Pooling Layer using DCCS [2]. DCCS is a new clustering framework that uses deep neural networks to learn the potential expression of images. Mutual information maximization was applied to embed relevant information in the latent representation. We trained the clustering model on four GPUs, and the batch-size was 128, the training epoch was 1000. In the initial experiment, we performed a clustering of the data into ten clusters, found that one cluster holds no patch, and another two clusters appeared highly adjacent distribution on the T-SNE diagram. Therefore, we repeated the training with cluster number set to eight, and the results showed that those patches could be classified into eight clusters, which we took as the final result.

***Grad-CAM:*** In the study, we also applied gradient-weighted class activation mapping (grad-CAM) to provide an insight into regions within each patch of the corresponding cluster that the neural network utilizes to generate predictions. We performed backward according to the predicted value of the model output, recorded the feature map and the corresponding gradient map on the last convolution layer of the CNN of the MVI-DL model. Then, we generated the activation map over the feature map combining with its weight, calculated from the average of each gradient map (the activation value was standardized by min-max).

**Simulation of the Clinical Application of the MVI-DL Model**

***Patients with Biopsies Only***

Considering that a large part of HCC patients could only acquire biopsy specimens, we simulated a clinical scenario where the tissue size of the WSIs were similar to liver biopsy specimens. Clinically, 18G needles are commonly used for liver biopsy. The internal diameter of the needle was 1.2 mm, while the length of punctured tissue was approximately 20 mm, so that the cross-sectional area of a liver biopsy was approximately 24 mm^2^. Therefore, we randomly selected one WSI for each patient in the FAHSYSU and DG-SD test sets, and then randomly sampled adjacent patches with a total area of 24 mm^2^ from each WSI as the one simulated biopsy. Clinically, three biopsies at most were routinely acquired for HCC patients. Therefore, we analysed the predictive performance of the MVI-DL model with one to three simulated biopsies.

[**Supplementary**](#_Toc3951) **References**

1 Ilse M, Tomczak JM, Welling M. Attention-based deep multiple instance learning [arXiv]. *arXiv* 2018:16.

2 Junjie Z, Donghuan L, Kai M*, et al*. Deep image clustering with category-style representation. In: Vedaldi A, Bischof H, Brox T*, et al.*, eds. 2020:54-70.

[**Supplementary**](#_Toc3951) **Tables**

**Table S1. Baseline characteristics of the patients in the FAHSYSU and DG-SD cohort.**

| **Variable** | | **FAHSYSU cohort** | |  | **DG-SD cohort** | ***P*-values** |
| --- | --- | --- | --- | --- | --- | --- |
|  |  | **Training set** | **Test set** |  | **External test set** |  |
| Age, No. (%) | | |  |  |  | 0.223 |
|  | ≤ 60 | 204 (76) | 60 (75) |  | 80 (67) |  |
|  | > 60 | 66 (24) | 20 (25) |  | 39 (33) |  |
| Gender, No. (%) | | |  |  |  | 0.798 |
|  | Male | 239 (88) | 69 (86) |  | 106 (89) |  |
|  | Female | 31 (12) | 11 (14) |  | 13 (11) |  |
| AFP, No. (%) | | |  |  |  | 0.569 |
|  | < 200 | 157 (58) | 43 (54) |  | 70 (61) |  |
|  | ≥ 200 | 113 (42) | 37 (46) |  | 44 (39) |  |
| Tumor number, No. (%) | | |  |  |  | 0.626 |
|  | single | 219 (81) | 67 (84) |  | 93 (78) |  |
|  | multiple | 51 (19) | 13 (16) |  | 26 (22) |  |
| Tumor size, No. (%) | | |  |  |  | 0.885 |
|  | < 5 cm | 123 (46) | 39 (49) |  | 54 (47) |  |
|  | ≥ 5 cm | 147 (54) | 41 (51) |  | 61 (53) |  |
| BCLC, No. (%) | | |  |  |  | 0.339 |
|  | 0-A | 178 (66) | 59 (74) |  | 79 (72) |  |
|  | B-C | 91 (34) | 21 (26) |  | 31 (28) |  |
| Edmondson grade, No. (%) | | |  |  |  | 0.009 |
|  | 1-2 | 143 (53) | 48 (60) |  | 83 (70) |  |
|  | 3-4 | 126 (47) | 32 (40) |  | 36 (30) |  |
| Tumor encapsulation, No. (%) | | |  |  |  | 0.289 |
|  | No | 21 (8) | 9 (11) |  | 6 (5) |  |
|  | Yes | 249 (92) | 71 (89) |  | 112 (95) |  |
| MVI status, No. (%) | | |  |  |  | 0.015 |
|  | No | 135 (50) | 35 (44) |  | 75 (63) |  |
|  | Yes | 135 (50) | 45 (56) |  | 44 (37) |  |

AFP, alpha fetoprotein; BCLC, Barcelona Clinic Liver Cancer.

**Table S2. Data used to train the segmentation network.**

|  | **WSIs** | **Patches (tumor)** | | |
| --- | --- | --- | --- | --- |
|  |  | **5×** | **10×** | **20×** |
| Training set | 682 | 47,777 | 190,899 | 735,675 |
| Validation set | 292 | 20,476 | 81,814 | 315,290 |

WSI, whole slide image.

**Table S3. Data used to train and test the MVI-DL model.**

|  | | **Patients** | **WSIs** | **Patches (Tumor)** | | |
| --- | --- | --- | --- | --- | --- | --- |
|  |  |  |  | **5×** | **10×** | **20×** |
| FAHSYSU cohort | |  |  |  |  |  |
|  | Training set | 270 | 2284 | 81,897 | 671,702 | 1,761,458 |
|  | Test set | 80 | 598 | 22,924 | 181,757 | 484,265 |
| DG-SD cohort | | 120 | 504 | 15,083 | 184,237 | 694,456 |

WSI, whole slide image.

**Table S4.** **Predictive performance of the MVI-DL model evaluated on the FAHSYSU and DG-SD test set.**

|  | | **AUC (95% CI)** | **Accuracy (95% CI)** | **Sensitivity (95% CI)** | **Specificity (95% CI)** |
| --- | --- | --- | --- | --- | --- |
| FAHSYSU test set | |  |  |  |  |
|  | 5× | 0.837 (0.814-0.860) | 0.808 (0.797-0.819) | 0.932 (0.916-0.948) | 0.647 (0.634-0.660) |
|  | 10× | 0.886 (0.864-0.908) | 0.825 (0.803-0.847) | 0.911 (0.875-0.947) | 0.714 (0.677-0.751) |
|  | 20× | 0.849 (0.811-0.887) | 0.784 (0.674-0.894) | 0.690 (0.627-0.753) | 0.906 (0.874-0.938) |
|  | MVI-DL model | 0.904 (0.888-0.920) | 0.833 (0.816-0.850) | 0.926 (0.894-0.958) | 0.710 (0.650-0.770) |
| DG-SD test set | |  |  |  |  |
|  | 5× | 0.843 (0.806-0.880) | 0.768 (0.758-0.778) | 0.850 (0.766-0.934) | 0.710 (0.640-0.780) |
|  | 10× | 0.855 (0.832-0.878) | 0.773 (0.747-0.799) | 0.830 (0.794-0.866) | 0.681 (0.664-0.698) |
|  | 20× | 0.825 (0.784-0.866) | 0.748 (0.736-0.760) | 0.683 (0.646-0.720) | 0.793 (0.731-0.855) |
|  | MVI-DL model | 0.871 (0.837-0.905) | 0.791 (0.761-0.821) | 0.900 (0.856-0.944) | 0.698 (0.638-0.757) |

AUC, area under the receiver operating characteristic curve; CI, confidence interval.

**Table S5. Univariate and multivariate analysis of factors associated with MVI in the training set.**

| **Variables** | **Univariable** | | |  | **Multivariable** | | |
| --- | --- | --- | --- | --- | --- | --- | --- |
|  | **OR** | **95% CI** | ***P*-values** |  | **OR** | **95% CI** | ***P*-values** |
| Age (≤ 60 vs > 60) | 0.85 | 0.49-1.48 | 0.571 |  | - | - | - |
| Gender (Female vs Male) | 0.60 | 0.28-1.28 | 0.185 |  | - | - | - |
| AFP (≥ 200 vs < 200) | 3.41 | 2.05-5.67 | < 0.001 |  | 2.55 | 1.46-4.46 | 0.001 |
| Tumor number (Multiple vs Single) | 1.41 | 0.76-2.60 | 0.278 |  | - | - | - |
| Tumor size (≥ 5 cm vs < 5 cm) | 3.55 | 2.14-5.87 | < 0.001 |  | 3.22 | 1.86-5.56 | < 0.001 |
| BCLC (B-C vs 0-A) | 2.66 | 1.57-4.50 | < 0.001 |  | 1.74 | 0.96-3.12 | 0.066 |
| Edmondson grade (3-4 vs 1-2) | 3.16 | 1.92-5.21 | < 0.001 |  | 2.58 | 1.49-4.48 | 0.001 |
| Tumor encapsulation (Yes vs No) | 2.12 | 0.83-5.42 | 0.118 |  | - | - | - |
| Prediction score | 1.08 | 1.06-1.10 | < 0.001 |  | 1.07 | 1.05-1.09 | < 0.001 |

AFP, alpha fetoprotein; BCLC, Barcelona Clinic Liver Cancer; OR, odds ratio; AUC, area under the receiver operating characteristic curve; CI, confidence interval.

**Table S6.** **Performance of the MVI-DL model in application to two simulated clinical scenarios.**

|  | | **AUC (95% CI)** | **Accuracy (95% CI)** | **Sensitivity (95% CI)** | **Specificity (95% CI)** |
| --- | --- | --- | --- | --- | --- |
| FAHSYSU test set | |  |  |  |  |
|  | One WSI | 0.875 (0.855-0.895) | 0.814 (0.738-0.890) | 0.793 (0.746-0.840) | 0.787 (0.758-0.816) |
|  | One biopsy | 0.843 (0.791-0.899) | 0.739 (0.669-0.810) | 0.849 (0.755-0.943) | 0.594 (0.476-0.713) |
|  | Two biopsies | 0.868 (0.832-0.904) | 0.779 (0.722-0.836) | 0.891 (0.825-0.957) | 0.631 (0.532-0.730) |
|  | Three biopsies | 0.879 (0.853-0.906) | 0.787 (0.743-0.830) | 0.903 (0.851-0.955) | 0.633 (0.549-0.717) |
| DG-SD test set | |  |  |  |  |
|  | One WSI | 0.837 (0.800-0.874) | 0.756 (0.669-0.843) | 0.648 (0.592-0.704) | 0.847 (0.785-0.909) |
|  | One biopsy | 0.803 (0.758-0.839) | 0.708 (0.645-0.771) | 0.648 (0.556-0.739) | 0.742 (0.650-0.835) |
|  | Two biopsies | 0.831 (0.805-0.857) | 0.730 (0.685-0.775) | 0.617 (0.559-0.674) | 0.795 (0.725-0.863) |
|  | Three biopsies | 0.841 (0.828-0.853) | 0.786 (0.763-0.809) | 0.645 (0.611-0.679) | 0.866 (0.837-0.895) |

AUC, area under the receiver operating characteristic curve; CI, confidence interval; WSI, whole slide image.

[**Supplementary**](#_Toc3951) **Figure Legends**

**Fig. S1. Illustration of the MVI-DL model architecture.** Patch bags which contained *N* patches (512×512 pixel) tiled from the tumor areas of the WSIs were fed into the pretrained Inception-v4 network along with the ground truth labels to extract the features [N, M] after data augment. Two linear layers activated by tanh and sigmoid function further transformed these features into a low-dimensional embedding, and the product of which passed through the attention liner layer and obtained an attention score for each patch [N, 1] by softmax. Then, the MIL Pooling layer performs max/average operation over features with their scores to obtain the aggregated features [1, M]. Finally, the MVI-DL generated the probability of the WSI through the fully connected layer.

**Fig. S2. Learning curves of the MVI-DL prediction model. (a).** The lines represent the AUCs of the MVI-DL prediction model for the training set (blue) and the validation set (pink). (**b).** The lines represent the loss of the MVI-DL prediction model for the training set (blue) and the validation set (pink).

**Fig. S3. Performance of the segmentation model in the classification of tumor and peri-tumor tissue. (a).** An example for manually annotated WSI. The red area was annotated as definite tumor tissue, and the green area was definite peri-tumor tissue. (**b).** Segmentation map of the automatic segmentation model output under 10× magnification scale. (**c).** Confusion matrices of the classification results under 5×, 10×, 20× and 40× magnification scale, respectively. (**d).** AUCs of the segmentation model in classifying tumor and peri-tumor on the validation set under different magnifications.

**Fig. S4. Performances of the MVI-DL model on the test sets.** Kaplan-Meier curves for OS analysis of the patients stratified by the MVI-DL model in the FAHSYSU (**a**), DG-SD (**b**) and TCGA (**c**) test sets. OS, overall survival.

**Fig. S5. Development and comparison of the clinical model, MVI-DL model and the combined clinical-MVI-DL model.** Comparison of the AUCs of the three models in the FAHSYSU (**a**) and DG-SD (**b**) test sets. ns, *P* > 0.05; *, 0.05 > *P* > 0.01; **, 0.01> *P* >0.001; ***, *P* <0.001.

**Fig. S6. The represented patches of each cluster and the pathological features of predictive clusters reviewed by the pathologist.**

**Fig. S7. Variation tendency of the predictive performance among different number of the WSIs per patient input into the MVI-DL model.** The maximum number of the WSIs randomly selected from each patient for analysis is the median number of the total WSIs per patient from the FAHSYSU test and DG-SD test cohort. Error bars is represented by standard deviation.

**Fig. S8. Simulation clinical scenarios of biopsy.** The AUCs of the MVI-DL model predicted with an increasing length of biopsy tissue in the FAHSYSU (**a**) and DG-SD (**b**) test set.
