# Supplementary figures and images for: Deep learning for evaluation of microvascular invasion in hepatocellular carcinoma from tumor areas of histology images
Source: Hepatol Int. 2022 Mar 28;16(3):590–602. doi: 10.1007/s12072-022-10323-w (PMC9174315; doi:10.1007/s12072-022-10323-w)

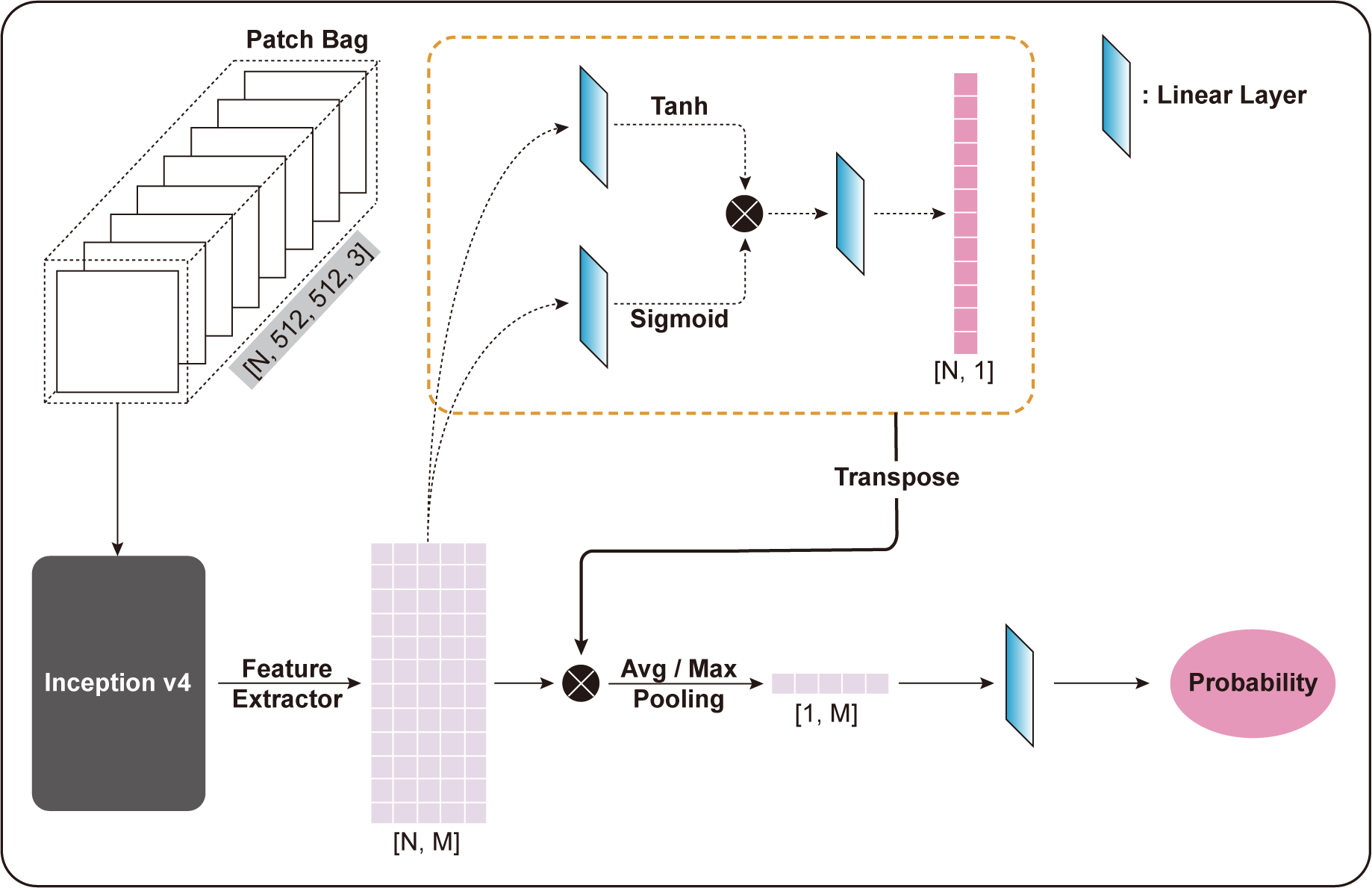

Supplement: Supplementary file 2 — Supplementary file2 (TIF 685 KB) [file 12072_2022_10323_MOESM2_ESM.tif]

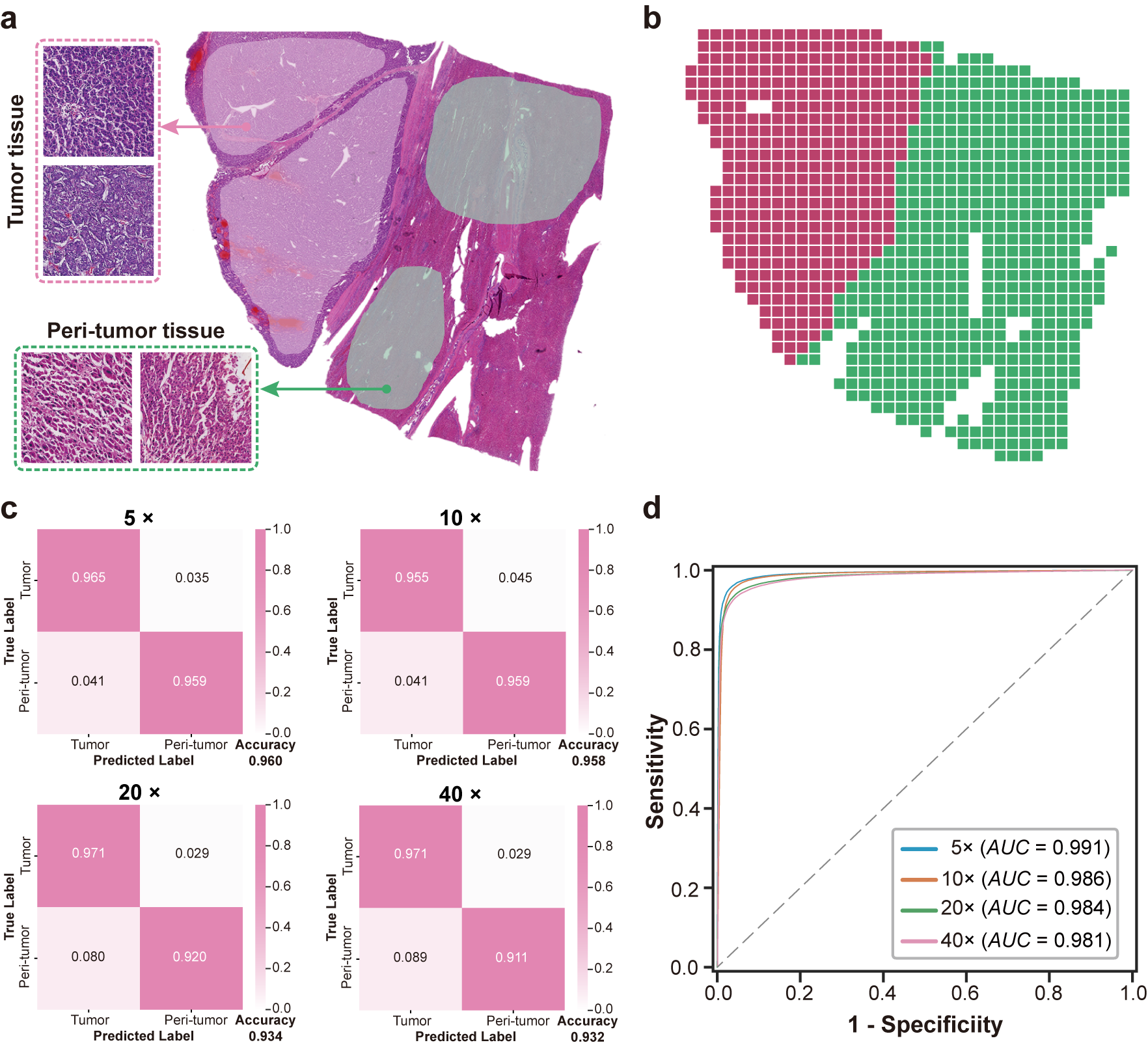

Supplement: Supplementary file 4 — Supplementary file4 (TIF 3592 KB) [file 12072_2022_10323_MOESM4_ESM.tif]

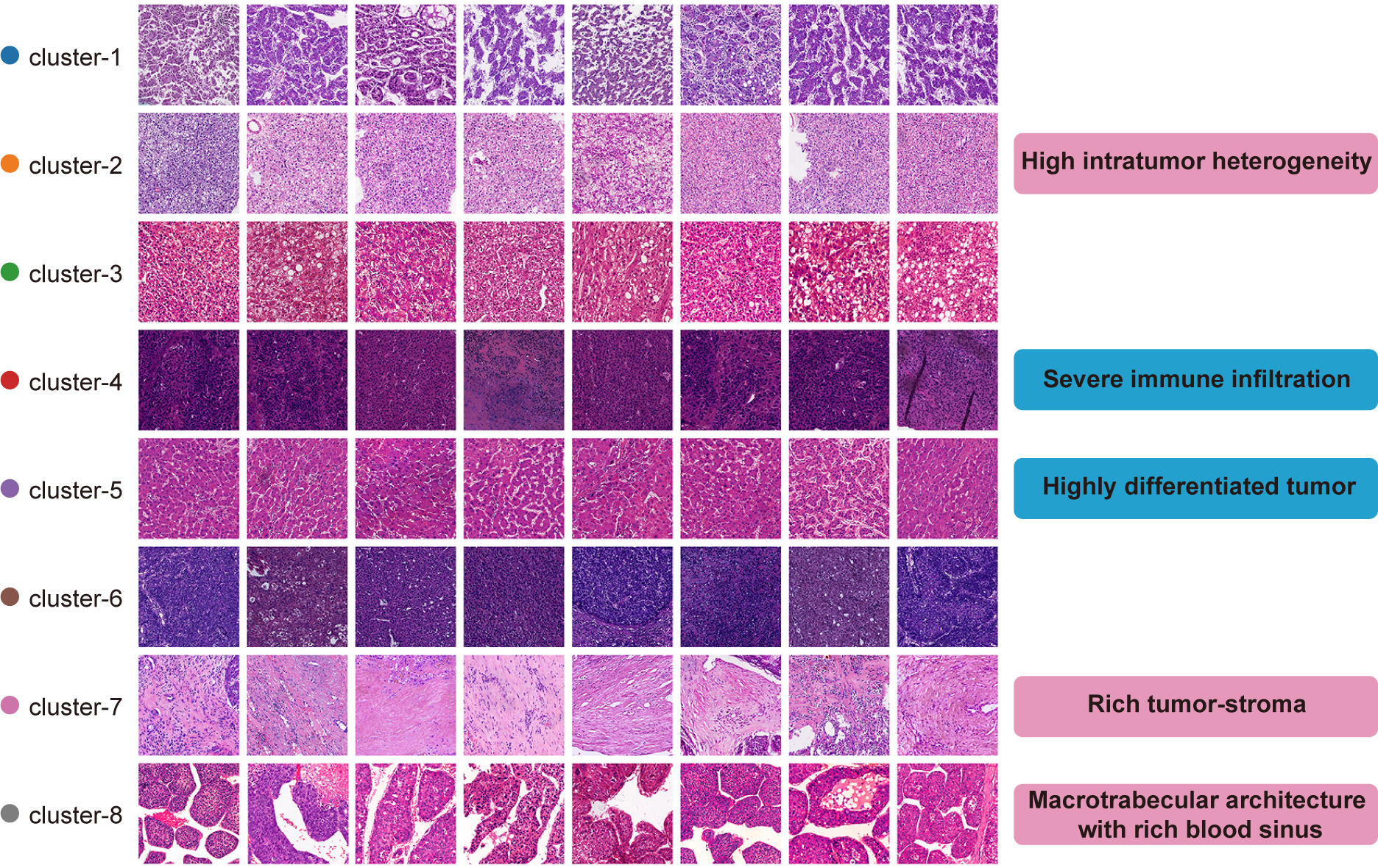

Supplement: Supplementary file 7 — Supplementary file7 (TIF 8307 KB) [file 12072_2022_10323_MOESM7_ESM.tif]
